# Fluctuation in anti-cyclic citrullinated protein antibody level predicts relapse from remission in rheumatoid arthritis: KURAMA cohort

**DOI:** 10.1186/s13075-020-02366-x

**Published:** 2020-11-12

**Authors:** Koichi Murata, Hiromu Ito, Motomu Hashimoto, Kosaku Murakami, Ryu Watanabe, Masao Tanaka, Wataru Yamamoto, Shuichi Matsuda

**Affiliations:** 1 Department of Advanced Medicine for Rheumatic Diseases, Kyoto University Graduate School of Medicine, 54 Kawahara-cho, Shogoin, Sakyo, Kyoto, 606-8507 Japan; 2 Department of Orthopaedic Surgery, Kyoto University Graduate School of Medicine, Sakyo, Kyoto, Japan; 3 Department of Rheumatology and Clinical Immunology, Kyoto University Graduate School of Medicine, Sakyo, Kyoto, Japan; 4 Department of Health Information Management, Kurashiki Sweet Hospital, Nakasho, Kurashiki, Japan

**Keywords:** Anti-citrullinated protein/peptide antibody, Biomarker, Relapse, Remission, Rheumatoid arthritis

## Abstract

**Background:**

The positivity of anti-citrullinated protein/peptide antibodies (ACPAs) is a clinically useful diagnostic and prognostic marker in rheumatoid arthritis (RA). However, the significance of ACPA titer and its fluctuation remain unclear. This study aimed to assess the role of ACPA titer and its fluctuation on disease activity and the prognosis of RA.

**Methods:**

Data obtained from the Kyoto University Rheumatoid Arthritis Management Alliance (KURAMA) cohort was analyzed. Patients whose ACPA was measured at least twice between 2011 and 2019 and whose ACPA was positive at least once were included in this study. The association between the clinical variable and ACPA titer or its change was investigated.

**Results:**

ACPA titer was measured in a total of 3286 patients, 1806 of whom were ACPA-positive at least once. Among them, the ACPA titer level was measured more than once in 1355 patients. Very weak correlation was observed between the ACPA titer level and disease activity. Additionally, there was no trend in the fluctuation of ACPA titer level in each patient; ACPA titer level fluctuated in some patients, but not in others. Patients with high variable levels of ACPA titer were more likely to relapse from remission. In the analysis of two consecutive ACPA measurements, the titer changes predicted the relapse from remission within a year of the second measurement.

**Conclusions:**

The ACPA titer level fluctuated in some patients. Very weak correlation was observed between the ACPA titer level and disease activity. Fluctuation in ACPA titer level predicted relapse from remission in patients with RA.

**Supplementary information:**

The online version contains supplementary material available at 10.1186/s13075-020-02366-x.

## Introduction

Rheumatoid arthritis (RA) is a chronic inflammatory disease with an estimated prevalence of 0.5% to 1% in adults, causing more disability, joint damage, deterioration of quality of life, and premature mortality compared with that in the general population [1]. RA identification at the initial presentation and treatment initiation at an earlier stage can affect the disease course, prevent the development of joint erosions or retard the progression of erosive disease [2]**.**

Anti-citrullinated protein/peptide antibodies (ACPAs) are sensitive and highly specific biomarkers for the diagnosis of RA that are present years before the onset of clinical RA [3]. Commercial assays use a synthetic cyclic citrullinated protein (CCP) as the antigen to detect ACPAs. Anti-CCP2 assays have reported a high specificity of greater than 95% and sensitivity of up to 70% in RA [4]. ACPA positivity has also been associated with severe, erosive disease. Currently, assessment of ACPA is used as a prognostic marker of RA, as well as a diagnostic marker [5].

Despite the association between ACPA and RA etiology, studies documenting the relationship between fluctuation in autoantibody level and disease activity have been conflicting [6]. In other autoimmune diseases, there is a link between autoantibodies and disease severity, and autoantibodies levels are measured [7–10]. Although it is currently known that the positivity of ACPA is a clinically useful diagnostic and prognostic marker, the significance of ACPA titer and its fluctuation is not yet elucidated. Thus, we measured the ACPA titer at least once a year and assessed the significance of ACPA titer level and its changes on disease activity and the prognosis of RA.

## Materials and methods

### Patients

The analyses in the current study were conducted using the Kyoto University Rheumatoid Arthritis Management Alliance (KURAMA) cohort database, with approval from the ethics committee of Kyoto University Hospital. The KURAMA cohort was established in May 2011 at the Center for Rheumatic Disease at Kyoto University Hospital with the aims of providing strict control of RA and using patient clinical and laboratory data for clinical investigations [11–13]. Clinical, biological and functional data were recorded at baseline and every visit.

Patients who were diagnosed as RA between 2011 and 2019, registered in this cohort, measured ACPA titer more than once and were positive for ACPA at least once were included in this study. During this period, a total of 2131 RA patients were registered, 1549 patients were measured for ACPA more than once, and 1355 patients were positive for ACPA at least once. All patients fulfilled the American College of Rheumatology revised criteria in 1987 [14] or the American College of Rheumatology (ACR)/European League Against Rheumatism (EULAR) criteria in 2010 [15].

The laboratory and clinical variables were extracted from the KURAMA cohort database at the time of examination. These data included age, sex, disease duration, swollen and tender joint counts, physician’s global assessment of RA activity, patient’s global assessment of RA activity, C-reactive protein (CRP) concentration, erythrocyte sedimentation rate (ESR), and the titers of rheumatoid factor (RF). The Disease Activity Score 28-joint count (DAS28) with ESR, clinical disease activity index (CDAI), and a simplified disease activity index (SDAI) were calculated. ACPA was measured using the ARCHITECT anti-CCP assay (Abbott Japan, Tokyo, Japan). Autoantibody titer levels were considered positive at greater than 15 IU/mL for RF and at greater than 4.5 U/mL for ACPA.

### Statistical analysis

All statistical analyses were conducted using the JMP Pro 13 software (SAS Institute Inc., Cary, NC), employing Fisher’s exact test for the comparison of categorical data. One-way analysis of variance or Kruskal–Wallis test was employed to compare the three groups, as appropriate. Pearson’s correlation coefficient (*r*) was used to measure the association between ACPA titer and the disease activity. The Cochran–Armitage trend test was employed to assess the trends of the relapse rate against the ACPA titer change. Logistic regression analysis was used to assess the factors predicting the relapse from the remission. Data are mean (standard deviation, SD) unless otherwise stated. A *p* values less than 0.05 were considered significant.

## Results

### Baseline demographic data, ACPA measurements, and change in ACPA titer level

Baseline data for all patients are presented in Table 1. Of the 1355 ACPA-positive patients whose ACPA titer level was measured more than once, 71.1% were women and 18.2% were smokers. The mean time from symptom onset to initial ACPA measurement was 1.8 months. RF positivity was 88.1%. At the time of the initial measurement, 4.1% of patients were prescribed methotrexate (MTX); 63.4% were prescribed MTX after 3 months of initial measurement. Prednisolone (PSL) was used by 2.2% of patients at the baseline and 30.8% of patients 3 months following the initial measurement. A total of 2.2% of the patients were prescribed with biological disease-modifying anti-rheumatic drugs (bDMARDs) or targeted synthetic disease-modifying anti-rheumatic drugs (tsDMARDs) at the baseline; 29.2% of the patients were treated with bDMARDs and tsDMARDs 3 months following the initial measurement.

**Table 1:** Baseline demographic and disease characteristics

	Total (*n* = 1355)	Change in ACPA titer level (maximum/minimum)			*P* value
		< 3 (*n* = 763)	3.1–5 (*n* = 297)	> 5 (*n* = 295)	
Age, years, mean (SD)	60.0 (13.8)	59.7 (14.5)	59.6 (13.3)	61.0 (12.2)	0.35
Female, *n* (%)	963 (71.1)	610 (80.0)	248 (83.5)	241 (81.7)	0.39
BMI, mean (SD)	21.7 (3.7)	21.7 (3.9)	21.7 (3.3)	21.7 (3.5)	0.99
Smoker (ever), *n* (%)	246 (18.2)	146 (19.1)	47 (15.8)	53 (18.0)	0.45
Disease duration, months, mean (SD)	1.8 (5.4)	1.8 (6.0)	1.8 (5.0)	1.6 (4.0)	0.85
Observational period (months), mean (SD)	4.6 (2.4)	45.7 (28.7)	65.5 (22.6)	68.7 (22.9)	< 0.001
Number of ACPA measurements, mean (SD)	5.3 (2.6)	4.6 (2.4)	6.1 (2.3)	6.5 (2.5)	< 0.001
RF (%)	88.1	86.7	89.9	89.9	0.37
RF, IU/ml, mean (SD)	150.4 (242.5)	155.1 (260.4)	148.4 (238.8)	140.6 (196.0)	0.77
ACPA (max), U/ml, mean (SD)	392.8 (579.7)	290.9 (365.0)	435.5 (499.5)	613.3 (934.6)	< 0.001
ACPA (min), U/ml, mean (SD)	137.8 (204.9)	170.1 (246.2)	112.6 (123.7)	71.9 (112.1)	< 0.001
ESR, mm/h, mean (SD)	31.8 (26.4)	29.7 (25.6)	33.0 (26.1)	35.7 (28.2)	< 0.05
CRP, mg/L, mean (SD)	12.4 (24.1)	10.5 (19.6)	11.3 (19.1)	18.3 (35.2)	< 0.001
CDAI, mean (SD)	11.7 (9.4)	11.2 (9.3)	11.8 (9.1)	12.9 (9.8)	0.06
SDAI, mean (SD)	13.2 (10.9)	12.5 (10.5)	13.1 (10.3)	14.9 (12.3)	0.05
DAS28 (ESR), mean (SD)	3.7 (1.4)	3.6 (1.4)	3.8 (1.4)	4.0 (1.4)	< 0.01
Tender joints, mean (SD)	2.4 (3.3)	2.3 (3.4)	2.4 (3.0)	2.6 (3.3)	0.1
Swollen joints, mean (SD)	2.5 (3.2)	2.5 (3.3)	2.4 (3.1)	2.7 (3.3)	0.23
MTX, *n* (%)	55 (4.1)	27 (2.0)	15 (5.1)	13 (4.4)	0.51
MTX, mg, mean (SD)	7.1 (2.8)	7.2 (2.5)	6.7 (3.3)	7.4 (3.2)	0.78
MTX (3 months) n (%)	859 (63.4)	477 (62.5)	201 (67.7)	181 (61.4)	0.21
MTX (3 months), mg, mean (SD)	8.2 (3.1)	8.1 (3.0)	8.1 (2.9)	8.3 (3.4)	0.72
Prednisolone, *n* (%)	30 (2.2)	16 (2.1)	7 (2.4)	7 (2.4)	0.95
Prednisolone, mg, mean (SD)	6.8 (8.6)	6.4 (8.7)	3.2 (2.3)	11.4 (11.3)	0.21
Prednisolone (3 months), *n* (%)	418 (30.8)	213 (27.9)	99 (33.3)	106 (35.9)	< 0.05
Prednisolone (3 months), mg, mean (SD)	6.7 (11.5)	5.9 (8.7)	7.1 (14.1)	8.0 (13.5)	0.27
bDMARDs or tsDMARDs, *n* (%)	30 (2.2)	11 (1.4)	10 (3.4)	9 (3.1)	0.09
bDMARDs or tsDMARDs (3 months), *n* (%)	395 (29.2)	200 (26.2)	96 (32.3)	99 (33.6)	< 0.05

One-way analysis of variance for continuous variables; Kruskal–Wallis test for CDAI, SDAI and DAS28-ESR; Fisher’s exact test for categorical variables among the three groups

*bDMARD* biological disease-modifying anti-rheumatic drug, *BMI* body mass index, *CDAI* clinical disease activity index, *CRP* C-reactive protein, *DAS28* Disease Activity Score 28-joint count, *ESR* erythrocyte sedimentation rate, *MTX* methotrexate, *RF* rheumatoid factor, *SD* standard deviation, *SDAI* simplified disease activity index, *tsDMARD* targeted synthetic disease-modifying anti-rheumatic drug

**Fig. 1:**
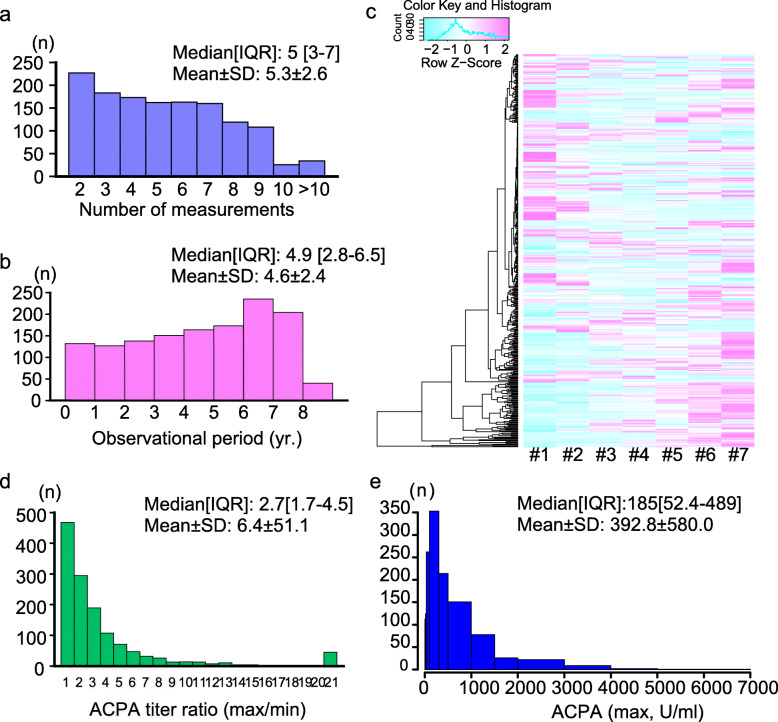
The number of anti-citrullinated peptide/protein antibody (ACPA) measurements and the fluctuation of ACPA titer level. **a** Distribution of the number of ACPA measurements in each patient. **b** Distribution of the follow-up period of each patient. **c** Heat map of the first seven ACPA titers for each patient whose titer levels were measured at least seven times. **d** The distribution of the ratio of maximum and minimum ACPA titer of each patient. **e** The distribution of the maximum ACPA titer of each patient. IQR, interquartile rage; SD, standard deviation

The median number of measurements of ACPA was 5 (interquartile range [IQR], 3–7) (Fig. 1a), and the average follow-up period was 4.6 years (Fig. 1b). Figure 1c presents the level change in the ACPA titer levels as an example of patients whose antibody level was measured seven times or more. Although ACPA titer levels gradually increased or decreased in some patients, the levels fluctuated up and down in numerous patients. The median change in ACPA titer level (ratio between maximum and minimum values) of each patient was 2.7 (IQR, 1.7–4.5) (Fig. 1d). The median of the maximum ACPA titer for each patient among the multiple measurements was 185 (IQR, 52.4–489) (Fig. 1e).

### Very weak correlation was observed between the ACPA titer level and disease activity in RA

**Fig. 2:**
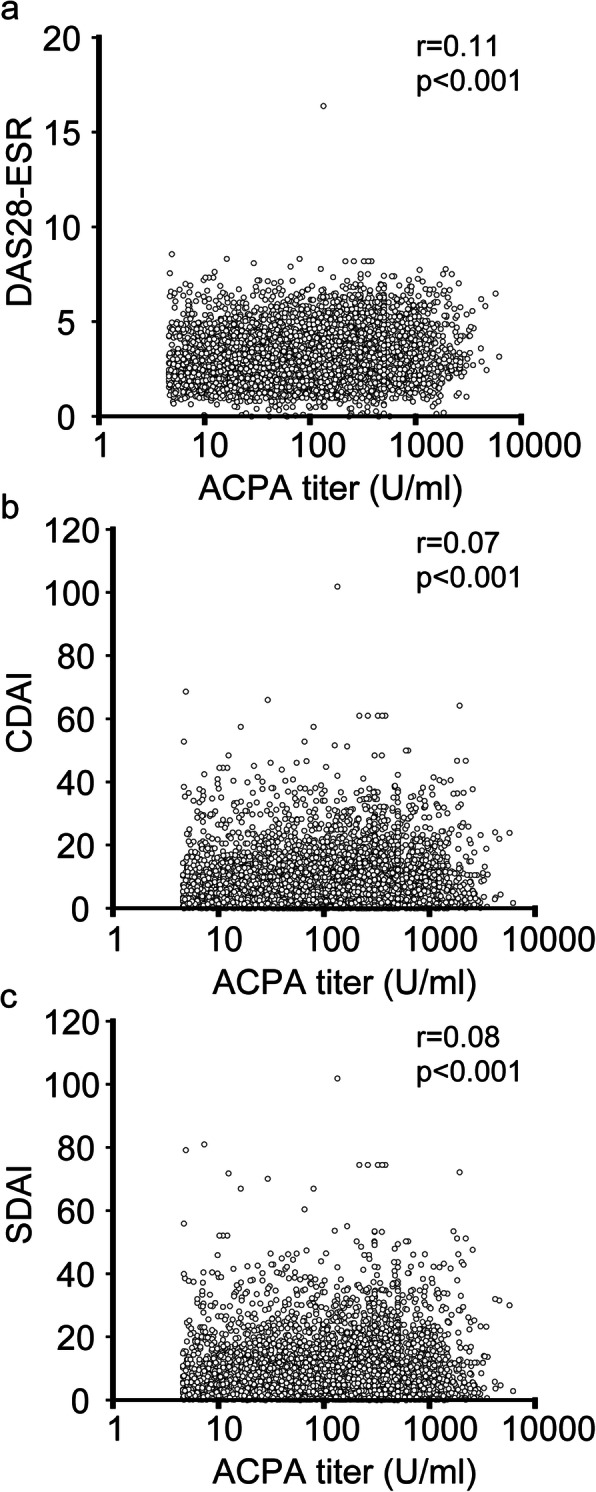
Relationship between ACPA titer and disease activity. The relationship between ACPA titer and DAS28-ESR (a, *n* = 5967), CDAI (b, *n* = 6342), and SDAI (c, *n* = 6042) was plotted. CDAI, clinical disease activity index; DAS28-ESR, Disease Activity Score 28-joint count; SDAI, simplified disease activity index

Figure 2 presents a plot of ACPA titer level and DAS28-ESR, CDAI, and SDAI when it was measured. As reported previously [16, 17], very weak correlations were observed between the ACPA titer levels and disease activity (*r* = 0.11, 0.07, and 0.08; *p* < 0.001, 0.001, and 0.001; respectively).

### Patients with high variable levels of ACPA titer were more likely to relapse from remission

As presented in Fig. 1d, the level of ACPA fluctuated in some patients, but not in others. Therefore, we divided the patients into groups based on the IQR in the fluctuation of ACPA titer levels (ratio of maximum to minimum): greater than fivefold, three to fivefold, and threefold or less.

There were no differences in age, female ratio, smoking rate, disease duration, RF positivity or usage of MTX, PSL, and bDMARDs among the three groups at baseline (Table 1). In patients with more than threefold change in ACPA, the follow-up period was longer, and the number of ACPA measurements was higher (*p* < 0.001 and 0.001, respectively). In these patients, the maximum ACPA titer was higher, and the minimum ACPA was smaller (*p* < 0.001 and 0.001, respectively). ESR, CRP, and DAS28-ESR were also higher in patients with high fluctuation in ACPA titer level (*p* < 0.05, 0.001 and 0.01, respectively). No difference was observed in tender or swollen joint counts (*p* = 0.1 and 0.23, respectively). In addition, there was no difference in the rate of MTX use 3 months following the initial ACPA measurement (*p* = 0.72), but the patients with large ACPA titer change were more frequently prescribed PSL and bDMARDs 3 months following the initial measurement (*p* < 0.05 and 0.05, respectively).

**Fig. 3:**
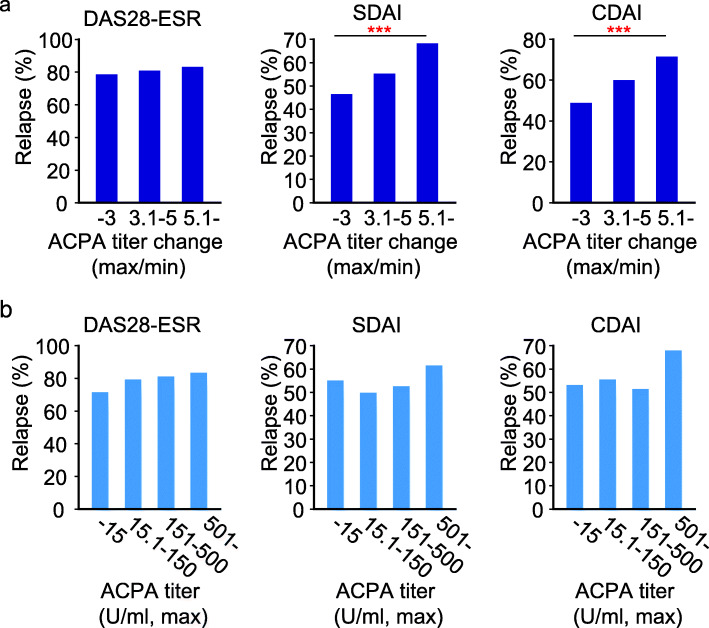
The relapse rate from remission in relation to ACPA titer level and its change. **a** The relationship between the ratio of maximum and minimum ACPA titer and the rate of relapse in patients who achieved remission during the follow-up period. **b** The relationship between the maximum ACPA titer and relapse rate. The Cochran–Armitage trend test was used to assess the trend. ***P < 0.001

The relapse rate was calculated in patients who achieved DAS28-ESR, CDAI, and SDAI remission at least once during the observational period in each group (*n* = 866, 687, and 669, respectively). Interestingly, higher probabilities of relapse from DAS28-ESR, SDAI, or CDAI remission were observed in patients who had experienced high fluctuation in ACPA titer levels (*p* = 0.05, < 0.001, and < 0.001, respectively, Fig. 3a).

Moreover, we calculated the probabilities of relapse from remission after dividing the patients into the three groups according to the ACPA maximum titer level of each patient. There were no statistically significant associations between the ACPA titer level and the rate of relapse from DAS28-ESR, SDAI, and CDAI remission (*p* = 0.20, 0.13, and 0.05, respectively, Fig. 3b).

To date, several factors have been proposed as predictors for sustained remission, including sex, short disease duration, not smoking, less tender joint count (TJC) at baseline, RF positivity, and absence of radiographic damage at baseline and initial treatment [18]. Thus, we investigated whether these factors could contribute to the relapse from the remission. Univariate analysis showed that disease duration, RF positivity, prednisolone usage, b/tsDMARDs usage, or MTX usage was not associated with the relapse (Supplementary Table 1). Smoking and TJC at baseline were associated with the relapse from DAS28-ESR remission (*p* < 0.01 and 0.01, respectively). Age, the radiographic damage at baseline, the prednisolone usage, and ACPA fluctuation were associated with the relapse from SDAI (*p* < 0.001, 0.05, 0.001, and 0.001, respectively). Age, the prednisolone usage, and ACPA fluctuation were associated with the relapse from CDAI (*p* < 0.001, 0.01, and 0.001, respectively). Multiple regression analysis showed that ACPA fluctuation and age were associated with the relapse from SDAI remission (*p* < 0.001 and 0.01, respectively) and CDAI remission (*p* < 0.001 and 0.01, respectively, Supplementary Table 2). This result further indicates that the change in the ACPA titer is closely associated with the relapse from remission.

### ACPA titer changes predicted relapse from remission within a year

**Fig. 4:**
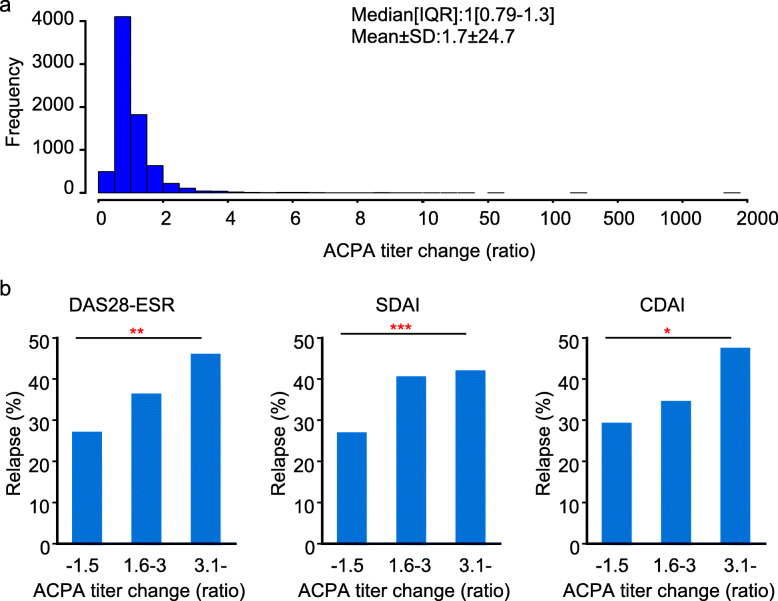
The relapse rate from remission in relation to the change in the consecutive ACPA titer level. **a** The ratio of consecutive ACPA titer was calculated, and the distribution is presented. **b** The relationship between the ratio of consecutive ACPA titer and the rate of relapse within 1 year from the second measurement in patients who achieved remission in two consecutive measurements. The Cochra0n–Armitage trend test was used to assess the trend. ***P < 0.001

Next, we investigated whether the changes in the consecutive ACPA titer levels can be a prognostic marker in RA. The median change in the consecutive ACPA titer levels (ratio) of each patient was 1 (IQR, 0.79–1.3) (Fig. 4a). This result suggests the ACPA titer did not change on average with an average follow-up period of 1 year. However, the ACPA titer changed in certain percentage of patients. Therefore, we divided the patients who achieved remission at the two consecutive measurements of ACPA into three groups: patients with ACPA titer level that increased greater than 3-fold, 1.5- to 3-fold, and others (Table 2). We then calculated the rate of relapse from remission within 1 year from the second ACPA measurement. Patients with a large increase in ACPA titer level had a higher rate of relapse from DAS28-ESR, SDAI, or CDAI remission (*p* < 0.001, 0.001, 0.001, respectively, Fig. 4b), whereas no differences were observed in RF positivity, CRP, and the rate of MTX, PSL, or bDMARDs users among the three groups (Supplementary Table 3).

**Table 2:** Baseline demographic and disease characteristics of patients who achieved remission in the consecutive measurement

	Remission		
	DAS28-ESR (*n* = 1160)	CDAI (*n* = 930)	SDAI (*n* = 946)
RF+, %	82.9	83.8	83.8
RF, average, IU/ml, mean (SD)	71.4 (100.2)	88.7 (189.0)	86.6 (183.3)
ESR, mm/h, mean (SD)	12.2 (8.5)	17.8 (14.4)	17.4 (13.9)
CRP, mg/L, mean (SD)	1.7 (3.9)	2.5 (8.4)	1.8 (4.0)
Measurement interval, mo., mean (SD)	12.2 (6.5)	12.4 (6.3)	12.4 (6.3)
MTX usage, %	61.3	61.9	61.4
MTX, mg, mean (SD)	7.6 (3.0)	7.5 (2.8)	7.5 (2.9)
Prednisolone usage, %	12.5	9.9	9.1
Prednisolone, mg, mean (SD)	4.1 (7.8)	4.7 (10.1)	4.5 (10.0)
bDMARD or tsDMARD usage, %	44.3	43.1	43.7
TNFα-i, %	23.7	25.8	26.5
IL6-i, %	18.6	10.3	10.1
CTLA4-Ig, %	3.8	6.4	6.4
JAK-i, %	0.1	0.6	0.7

*IL6-i* interleukin 6 inhibitor, *JAK-i* Janus kinase inhibitor, *TNFα-i* tumor necrosis factor inhibitor

## Discussion

ACPAs have been shown to play an important pathogenic role, both at the onset and during the course of the disease. They appear years before the clinical onset of RA, and their positivity is specific in the diagnosis of RA. Furthermore, ACPA-positive RA patients have a more aggressive clinical course with increased risk of pulmonary and cardiovascular complications, as well as radiographic progression and osteoporosis [5]. Multiple pieces of evidence support the association between ACPA and RA pathogenesis [3, 5].

Despite the high predictive value for the development and prognosis of RA, the literature on the sequential testing of ACPA titer is limited. A recent study measured IgG, IgM, and IgA of anti-CCP2 at 4-month intervals over the first year of treatment in 381 seropositive RA patients. However, the study found that there was no association among autoantibody level and DAS, EULAR response, and long-term outcome, including drug-free remission or radiographic progression [6].

The ACPA titer level changes have also been documented after bDMARD usage. Treatment with etanercept or adalimumab was shown to decrease the ACPA titer level [5]. During treatment with rituximab, reduction in ACPA titer level was observed in patients with ACR20 response at 6 months [19]. A reduction in ACPA titer level and seroconversion was observed during treatment with abatacept [20, 21]. However, the number of patients in these studies is limited, and whether these changes in autoantibody levels are indicative of future disease course remains unknown.

Currently, accumulating evidence suggests the role of ACPA in the onset of RA rather than the progression [22]. In addition to the presence of ACPA several years before the disease onset, bone loss and lung disease were observed in patients with ACPA who had no clinical signs of RA [23, 24]. A high probability of progression to inflammatory arthritis is also reported if ACPAs were present [25]. Conversely, in the synovial tissue with chronic inflammation, synovial fibroblasts and tissue macrophages stimulate each other through the production of cytokines, such as TNF and granulocyte-macrophage colony-stimulating factor [26].

Considering this evidence, it is natural to focus on the patients in remission to elucidate the role of ACPA titer rather than on patients with high disease activity. The fact that there were no correlations between the disease activities, such as DAS28-ESR, and the ACPA titer (Fig. 2) supports this idea.

The ratio of the maximum titer to the minimum titer and the ratio of the consecutive level of ACPA antibody, rather than the absolute titer level of the antibody, were correlated with the possibility of relapse from remission (Figs. 3a and 4b). ACPA is known to recognize a wide variety of citrullinated proteins, including fibrin, fibrinogen, vimentin, alpha-enolase, type II collagen, peptidylarginine deiminase enzyme, enolase, and tenascin [5, 27]. The reason why the ratio was more clinically meaningful than the absolute value of the titer might be that the antibodies detected by the anti-CCP2 assay contained antibodies which recognized the antigens not related to the pathology of RA, or contained antibodies that had weak affinity to the antigen, and that the effect of the antibody concentration increase by such antibody might be diluted by calculating the ratio.

In addition, the epitope spreading of the ACPA occurs in the years preceding the onset of arthritis, and epitope expansion and ACPA titer increase can be observed just before the development of clinical RA [3, 28, 29]. Identifying the increase in the ACPA titer level is important to predict the appearance of arthritis. This is in line with the fact that relapse from remission was associated with the increase in ACPA ratio. The current findings have some limitations. First, this is an observational cohort. Although the treatment was provided based on a treat-to-target strategy according to the guidelines by ACR, EULAR, and Japan College of Rheumatology, there was room for the physician to select the treatment. RA patients with high levels of ACPA titers could undergo stronger treatment. However, it is unlikely that the physician treated the patients referencing the ACPA ratio because it is a new parameter. Second, the effects of other cofactors that may contribute to relapse were not fully considered. It is difficult to define and detect the weakened treatment in the retrospective observational study. But it is noteworthy that the ACPA fluctuation was associated with the relapse from the remission even if multivariate analysis is performed using the baseline factors as covariates. Third, the threshold of ACPA titer change ratio needs to be validated in the future. The mean ratio of ACPA titer change was 1.0 when the average measurement interval was 1 year (Fig. 4a) but increased to 2.7 during the average 4.6 years of follow-up (Fig. 1d). In other words, if the measurement interval of ACPA becomes longer, the range of fluctuation may increase in some patients. Thus, it is important to set an appropriate cutoff if the change in ACPA titer level is used as a prognostic marker of relapse. Fourth, the mechanism of the ACPA titer fluctuation is unknown. It is the focus of the future investigation whether specific DMARDs can suppress fluctuations. Considering that the ratio of titer change is more meaningful than the maximum titer of ACPA, it is necessary to clarify what ACPA recognizes, and how the ACPA titer change is involved in the pathological condition.

The strength of this study is that the ACPA titer was measured in a large number of patients over a long period of time. Most studies thus far have reported the result within a year or before and after using bDMARDs, but there are no reports showing the titer changes up to 8 years. In addition, we first identified the significance of the change in ACPA titer level, which had not been elucidated so far. This study is valuable because it added further indirect evidence that ACPA is involved in the pathogenesis of RA. Moreover, it revealed the significance of measuring the ACPA titer level periodically.

## Conclusions

The ACPA titer level fluctuates in some RA patients. Very weak correlation was observed between the ACPA titer level and disease activity. The fluctuation in ACPA titer level predicts relapse from remission in RA patients.

## Supplementary Information

**Additional file 1: Supplementary Table 1.** The univariate analysis of patients’ characteristic and the relapse from remission. Logistic regression analysis was used. RF, rheumatoid factor; TJC, tender joint count; MTX, methotrexate; DMARDs, disease modifying anti-rheumatic drugs; ACPA, anti-citrullinated peptide/protein antibody. **Supplementary Table 2.** The multivariate analysis of patients’ characteristic and the relapse from remission. Logistic regression analysis was used. **Supplementary Table3.** Detailed baseline demographic and disease characteristics of patients who achieved remission in the consecutive measurement. One-way ANOVA for continuous variables, Kruskal–Wallis test for CDAI, SDAI and DAS28-ESR, Fisher’s exact test for categorical variables among three groups.

## Data Availability

The dataset used and/or analyzed in the current study are available from the corresponding author on reasonable request.
