# Supplementary material for: Fluctuation in anti-cyclic citrullinated protein antibody level predicts relapse from remission in rheumatoid arthritis: KURAMA cohort
Source: Arthritis Res Ther. 2020 Nov 12;22:268. doi: 10.1186/s13075-020-02366-x (PMC7664066; doi:10.1186/s13075-020-02366-x)
Supplement: Supplementary file 1 — Additional file 1: Supplementary Table 1. The univariate analysis of patients’ characteristic and the relapse from remission. Logistic regression analysis was used. RF, rheumatoid factor; TJC, tender joint count; MTX, methotrexate; DMARDs, disease modifying anti-rheumatic drugs; ACPA, anti-citrullinated peptide/protein antibody. Supplementary Table 2. The multivariate analysis of patients’ characteristic and the relapse from remission. Logistic regression analysis was used. Supplementary Table3. Detailed baseline demographic and disease characteristics of patients who achieved remission in the consecutive measurement. One-way ANOVA for continuous variables, Kruskal–Wallis test for CDAI, SDAI and DAS28-ESR, Fisher’s exact test for categorical variables among three groups. [file 13075_2020_2366_MOESM1_ESM.docx]

|  | Univariate analysis | |  |  |  |  |  |  |  |
| --- | --- | --- | --- | --- | --- | --- | --- | --- | --- |
|  | DAS28-ESR remission | |  | SDAI remission | |  | CDAI remission | |  |
|  | OR | 95%CI | p-value | OR | 95%CI | p-value | OR | 95%CI | p-value |
| Age (/yr) | 1.0 | 0.98~1.01 | 1 | 0.98 | 0.97~0.99 | <0.001 | 0.98 | 0.97~0.99 | <0.001 |
| sex(female/male) | 0.99 | 0.6~1.5 | 0.96 | 1.05 | 0.7~1.6 | 0.78 | 1.4 | 0.9~2.1 | 0.12 |
| Disease duration (mo) | 0.97 | 0.9~1.0 | 0.09 | 1.0 | 0.97~1.0 | 0.91 | 1 | 0.97~1.03 | 0.92 |
| Smoker (ever) | 0.56 | 0.4~0.8 | <0.01 | 0.87 | 0.6~1.3 | 0.46 | 0.86 | 0.6~1.3 | 0.44 |
| Radiographic damage (baseline) | 1.2 | 0.7~2.2 | 0.43 | 1.8 | 1.0~3.1 | <0.05 | 1.5 | 0.9~2.7 | 0.14 |
| RF positivity (baseline) | 0.98 | 0.6~1.7 | 0.93 | 1.04 | 0.6~1.8 | 0.89 | 1.1 | 0.6~2.0 | 0.73 |
| TJC (baseline, /1 joint) | 1.1 | 1.0~1.2 | <0.01 | 0.6 | 0.2~2.1 | 0.42 | 0.96 | 0.9~1.0 | 0.18 |
| MTX usage (3 mo) | 1.1 | 0.8~1.6 | 0.67 | 1.2 | 0.8~1.6 | 0.4 | 1.2 | 0.9~1.7 | 0.26 |
| Prednisolone usage (3 mo) | 0.87 | 0.6~1.3 | 0.48 | 2.0 | 1.3~2.8 | <0.001 | 1.8 | 1.3~2.7 | <0.01 |
| b/tsDMARDs usage (3 mo) | 1.2 | 0.8~1.7 | 0.41 | 1.3 | 0.9~1.7 | 0.16 | 1.3 | 0.9~1.7 | 0.18 |
| ACPA fluctuation (> 3 folds) | 1.3 | 0.9~1.8 | 0.14 | 1.9 | 1.4~2.6 | <0.001 | 2.1 | 1.5~2.8 | <0.001 |
|  |  |  |  |  |  |  |  |  |  |
| Supplementary Table 1. |  |  |  |  |  |  |  |  |  |
| The univariate analysis of patients’ characteristic and the relapse from remission. | | | | |  |  |  |  |  |
| Logistic regression analysis was used. |  |  |  |  |  |  |  |  |  |
| RF, rheumatoid factor; TJC, tender joint count; MTX, methotrexate; DMARDs, disease modifying anti-rheumatic drugs; | | | | | | | |  |  |
| ACPA, anti-citrullinated peptide/protein antibody | | |  |  |  |  |  |  |  |

|  | Multivariate analysis | |  |  |  |  |  |  |  |
| --- | --- | --- | --- | --- | --- | --- | --- | --- | --- |
|  | DAS28-ESR remission | |  | SDAI remission | |  | CDAI remission | |  |
|  | OR | 95%CI | p-value | OR | 95%CI | p-value | OR | 95%CI | p-value |
| Age (/yr) |  |  |  | 0.27 | -0.03~0.005 | <0.01 | 0.26 | 0.11~0.62 | <0.01 |
| sex(female/male) |  |  |  |  |  |  |  |  |  |
| Disease duration (mo) |  |  |  |  |  |  |  |  |  |
| Smoker (ever) | 0.6 | 0.4~0.9 | <0.05 |  |  |  |  |  |  |
| Radiographic damage (baseline) |  |  |  |  |  |  |  |  |  |
| RF positivity (baseline) |  |  |  |  |  |  |  |  |  |
| TJC (baseline, /1 joint) | 5.8 | 1.8~18.9 | <0.01 |  |  |  |  |  |  |
| MTX usage (3 mo) |  |  |  |  |  |  |  |  |  |
| Prednisolone usage (3 mo) |  |  |  | 1.7 | 1.2~2.5 | <0.01 |  |  |  |
| b/tsDMARDs usage (3 mo) |  |  |  |  |  |  |  |  |  |
| ACPA fluctuation(> 3 folds) |  |  |  | 1.7 | 1.3~2.4 | <0.001 | 2 | 1.4~2.7 | <0.001 |
|  |  |  |  |  |  |  |  |  |  |
| Supplementary Table 2. |  |  |  |  |  |  |  |  |  |
| The multivariate analysis of patients’ characteristic and the relapse from remission. | | | | |  |  |  |  |  |
| Logistic regression analysis was used. |  |  |  |  |  |  |  |  |  |

|  | DAS28-ESR remission | | |  | CDAI remission | | |  | SDAI remission | | |  |
| --- | --- | --- | --- | --- | --- | --- | --- | --- | --- | --- | --- | --- |
|  | ≤ 1.5 fold | 1.5 - 3 fold | > 3 fold | p-Value | ≤ 1.5 fold | 1.5 - 3 fold | > 3 fold | p-Value | ≤ 1.5 fold | 1.5 - 3 fold | > 3 fold | p-Value |
|  | (n = 953) | (n = 181) | (n = 26) |  | (n = 762) | (n = 147) | (n = 21) |  | (n = 772) | (n = 155) | (n = 19) |  |
| RF+ (%) | 82.3 | 85.6 | 82.9 | 0.31 | 82.9 | 89.4 | 79.2 | 0.22 | 83.2 | 87.8 | 78.3 | 0.44 |
| RF, average, IU/ml, mean (S.D.) | 70.0 (96.2) | 79.2 (124.3) | 71.0 (54.1) | 0.48 | 82.0 (175.5) | 126.4 (255.0) | 77.0 (76.0) | <0.05 | 81.7 (172.6) | 113.0 (236.4) | 75.6 (84.8) | 0.12 |
| ESR, mm/h, mean (S.D.) | 12.0 (8.4) | 13.3 (8.9) | 11.8 (7.3) | 0.15 | 17.2 (13.6) | 21.3 (16.9) | 18.2 (18.7) | <0.05 | 16.9 (13.4) | 20.2 (16.3) | 15.0 (12.3) | <0.05 |
| CRP, mg/L, mean (S.D.) | 1.6 (3.1) | 2.2 (6.9) | 1.6 (1.9) | 0.18 | 2.5 (8.8) | 2.5 (5.3) | 4.0 (7.9) | 0.68 | 1.7 (3.8) | 2.3 (5.1) | 1.9 (2.2) | 0.27 |
| Interval, mo, mean (S.D.) | 12.0 (6.4) | 12.9 (5.0) | 17.8 (12.8) | <0.001 | 12.1 (0.2) | 13.5 (0.5) | 15.6 (1.3) | <0.001 | 12.1 (6.2) | 13.6 (6.2) | 15.7 (10.5) | <0.001 |
| MTX (%) | 61.5 | 62.2 | 50 | 0.5 | 62.9 | 58.1 | 54.2 | 0.17 | 62.1 | 59.3 | 52.2 | 0.28 |
| MTX, mg, mean (S.D.) | 7.6 (3.1) | 7.2 (2.7) | 6.5 (2.8) | 0.1 | 7.6 (2.9) | 7.2 (2.7) | 7.4 (1.9) | 0.56 | 7.6 (2.9) | 7.3 (2.7) | 7.3 (2.0) | 0.1 |
| Prednisolone (%) | 12.2 | 14.9 | 9.4 | 0.6 | 9.5 | 11.9 | 12.5 | 0.32 | 8.6 | 11.1 | 13 | 0.23 |
| Prednisolone, mg, mean (S.D.) | 3.3 (2.2) | 7.4 (17.7) | 5.7 (4.0) | <0.05 | 3.4 (2.4) | 10.2 (22.5) | 5.0 (3.0) | <0.05 | 3.4 (2.4) | 8.7 (22.2) | 5.0 (3.0) | 0.12 |
| bDMARDs (%) | 44.7 | 41.8 | 50 | 0.85 | 43.8 | 38.8 | 45.8 | 0.44 | 44.3 | 40.1 | 47.8 | 0.56 |
|  |  |  |  |  |  |  |  |  |  |  |  |  |
| Supplementary Table3. |  |  |  |  |  |  |  |  |  |  |  |  |
| Detailed baseline demographic and disease characteristics of patients who achieved remission in the consecutive measurement. | | | | | | | | | |  |  |  |
| One-way ANOVA for continuous variables, Kruska-Wallis test for CDAI, SDAI and DAS28-ESR, Fisher’s exact test for categorical variables among three groups. | | | | | | | | | | | |  |
